# Supplementary material for: Structural Insights into Common and Host-Specific Receptor-Binding Mechanisms in Algal Picorna-like Viruses
Source: Viruses. 2022 Oct 27;14(11):2369. doi: 10.3390/v14112369 (PMC9697754; doi:10.3390/v14112369)
Supplement: Supplementary file 1 [file viruses-14-02369-s001.zip › viruses-1969268-supplementary.pdf]

Parameter or statistic	Full CsfrRNAV	Empty CsfrRNAV
Data collection and processing		
Magnification	79,000	79,000
Voltage (kV)	300	300
Electron exposure (e^{-}/Å^2)	30	30
Defocus range (μm)	-0.10 to -4.00	-0.10 to -4.00
Pixel size (Å)	1.1041	1.1041
Symmetry imposed	I	I
Initial micrographs (No.)	10,310	10,310
Final micrographs (No.)	9,947	9,772
Initial particle images (No.)	174,942	152,127
Final particle images (No.)	3,984	1,302
Map resolution (Å)	3.0	3.1
FSC threshold	0.143	0.143
Map resolution range (Å)	2.696 to 3.406	2.4 to 3.588
Refinement		
Map sharpening *B* factor (Å^2)	91.6	82.9
Model composition		
Non-hydrogen atoms	6171	5689
Protein residues	785	718
B factors (Å^2)		
Protein (min./max./mean)	17.34/122.72/55.06	20.66/131.81/64.13
RMSD*		
Bond lengths (Å)	0.003	0.005
Bond angles (°)	0.680	0.627
Validation		
MolProbity score	2.27	1.88
Clashscore	5.86	7.52
Poor rotamers (%)	3.72	0
Ramachandran plot		
Favored (%)	90.99	92.39
Allowed (%)	9.01	7.61
Disallowed (%)	0	0
CC (mask)	0.88	0.88
EMRinger score	3.21	3.25
Data deposition		
EMDB	EMD-15823	EMD-15830
PDB	8B38	8B3J

*RMSD, root mean square deviation

Table S1. Cryo-EM data collection, refinement, and validation statistics.

Genus	Species	Virus name	Accession number	Abbreviation	Average pLDDT (%)	VP1 sequence used for Alphafold
Bacillarnavirus	*Chaetoceros socialis forma radians RNA virus 1*	Chaetoceros socialis f. radians RNA virus 01	AB469874	CsfrRNAV	N/A	N/A
Bacillarnavirus	*Chaetoceros tenuissimus RNA virus 01*	Chaetoceros tenuissimus RNA virus 01	AB375474	CtenRNAV01	82	GAQKLTEFVSDVTSEVVETPSSWDITRADALNPEADLGSFLSRPVQLARINWTNSSYLQTSLNVWSDFLTHPTIVKKLQNYSWLSGSLHVKLQINGGPFYFGKALASYTPYGFSAPTNISNNYVRNNSLARMSQRDHVILDPTECKGGTIVCPFVYPKPYLSLDTADLASLDGVGRVDIMSFEALQNTSADTSTPINISVYAWMEDVRLSGPTYLRSESKM
Bacillarnavirus	*Rhizosolenia setigera RNA virus 01*	Rhizosolenia setigera RNA virus 01	AB243297	RsRNAV01	80	DVSVNVGFNSEVPITHTEIKSAFDPLRSDGWDSKLELTSFLRRPVRIYEKTWTVGSFLRDDFFPWDAYLNTASIRRKLENYLYLRGSLKLQIFINGTQFHYGKGIVAYTPFGAAQHPFRTPGSAPVLDNVQYSQKPHVLLDPTDNVGGEMTLPFVFPHNWVRHTIQSDLQQLGQIDLSSFNKLGHSLGDVTSPVRIAIFAYMDDDVALSGSTQFSS
Kusarnavirus	*Astarnavirus*	Asterionellopsis glacialis RNA virus	AB973945	AglaRNAV	90	NSEPGLTMSESAPAVEQQITAFADQDAGWKTEIHGNYDATMDKVETSNSDLGSFLQRPIRQSVQTWLINQPLYYKFNPWKEFMENPFIRNKIANYELVRCKMHCKMVISGTKFHYGRALTSYNPLSGFDQVTVERNYLDQDLVQASQKPHFFLNPTKNTGGEICMPFFWEKNYLSLSDKDYENMGELTIKSFGNLLHANGGNDPVTVTIYLWAEDVVLTMPTSLNPI
Labyrnavirus	*Aurantiochytrium single-stranded RNA virus 01*	Aurantiochytrium single-stranded RNA virus 01	AB193726	ASSRNAV01	68	ESKEVGEMSMGGIDHTETHQNVQDHTGEASDRVTVGSSIQETGDGSGTQYSLNSFFERPVAIYDATWDSGTEYNVNFEPWDLWSKDTSVRAKLANYSYFKGTMHIKIATTGTPYHYGTIMASYQPYGVYNQNLIKYDEMMNATTPGPGPTLPAYKCYLSQAPGVAYIDVKENQPVELEIPFISHKPKFRLFNNSPDVITNATSFVDFKEAGELRLVTLNKLAVANDDFDSDVSLNIYAWVTDVELGNITATDMD
Locarnavirus	*Jericarnavirus B*	Marine RNA virus JP-B	EF198242	JP-B	84	QSSDIIEMTPESTGNTMQEENVGFDGQQDPILTIPNDLSRVQVDQSQNVTLGNFLKRPVQIATQTWTVGSTLDQLVDNFDPWHLYFNHAPIRRKLDNYYLVRCNLHLKFVVNASPFFYGCTLASYQPLTNFAPGFTPTGTAGQERTAYTQRPHIWIYPQDSQGGEMVLPFLYYKNWLDATSAVDLTNMGTLSFQSFGSLANANGATGDIEIIVYAWAEDIEVAGPTVALAV
Locarnavirus	*Sanfarnavirus 1*	Marine RNA virus SF-1	JN661160	SF-1	90	VQADVIPSVETGVTGDQRQTVTFLDTNPGDHAGIERSIDSSMTADQTPNADLREFFSRPVRIASFTWLESDAVGTSHTFNPWNLYFTDQRVKYKLNNFAFIQAELEIKILVNASPFYYGQMLCAYQPLPGLTPSTITNDTGTRYFIPYSQRPHVWLEPTGNKGGSLTLPFFYHKNWLNAQSAQDMTDMGQLTFLNYTTLASANGVAGSGVTVSVFASAKNVRLSGPSVGLAV
Locarnavirus	*Sanfarnavirus 2*	Marine RNA virus SF-2	KF412901	SF-2	84	VATNLPTTDFNDQVAGVTVSPDTNWAEADVGDANTLADLSGFLQRPVRIHTLNWLESDPVGYLGLNTIDPWSLFFSNAAIKRKIDNYAFLRATLKIKIIINSTPFNYGAAKVVYTPLWTTHGRIPTSTNALNLVPQSQLPGAWIFPQNSQGCELSLPFYKHENFIALTSATDMADMGRLSFLVYSALKSANGVTATGVSIQV[illegible]
Locarnavirus	*Sanfarnavirus 3*	Marine RNA virus SF-3	KF478836	SF-3	86	EKQSDESTPMETSSATNETTQETVSFMDEGTTYATGTAAAPPSSAPADALTGAELGDFLGRPAQIASFTWSQSDVVGTTRSYNVWQNFFNNAAIRRKLENFAWLRCDLKVKIMVNASPFYYGAMLATYQPLPNFSPSTIVNDTGTRYFIPLTQRPHAWIYPQNNEGAEMTLPFFWPKNWLSTLSNADFADMGTLSLVSFTDLLSANGATGSGATFTIYAWAENVTLNGPTCGLIM
Marnavirus	*Heterosigma akashiwo RNA virus*	Heterosigma akashiwo RNA virus	AY337486	HaRNAV	76	TCTPHLDEKLLTETLCNTLKFYDTSGLRENSHQTMSNGTERRPGEVDGDLASILSRPTRVFTDTWKPGTNFYQASNIWKSFLNDDTIVDKISHFARLRGKMVVRLLINGNSMYYGKLVMHYSPFAQVDDVYTVSSVPDPAEWIQIMQKPHVSFDATTTTGATMELPMLLPNDWIDLTDEDMIARLGTLYIHDLNTLEHANAGTDELTVTLVAWMEDVELYLPTSTSEI
Salisharnavirus	*Britarnavirus 4*	Marine RNA virus BC-4	MH171300	BC-4	89	ADGTTNDNSVMKISGAGKYENVCFSDQVDPYAYNVESIMDPTRKLQDTGDATLQNFFSRPIKIEQVEWATSSQLIFDVNPWEAYFGNTRVANRLSNFNLLRAKLHVKIVINGNGFQYGRAIASYLPFSIYDTLSTNAGLIRQDLVQASQQPHLFLDPTTSQGGEMTLPFYNYWNYSSIPDTQWNELGILQFRSINDLKHSNGASDVVTVSVFAWAEDVEMSVLTSVDQD
Salisharnavirus	*Palmarnavirus 473*	Marine RNA virus PAL473	KT727026	PAL473	87	FNVNINTDTKMMSSQNVTFKDQTPSYSYSVAANNDATFEVADRSDASLGEFFSRPVKIREFQWATTTTLFESFNPWTDYFTNPRVLNRITNFGLLRSKLCLKFVINGNGFHYGRIICSYIPLRDDDDFTVDRAFIPEDIVGASQRPHIYLDPTTSSGGTMCLPFFWYNNCLEVPNEEWSKMGTMIMHTMQSLQHANGANDSATISVFAWAEDVTLAVPTSSEPG
Sogarnavirus	*Britarnavirus 2*	Marine RNA virus BC-2	MG584188	BC-2	86	GTIQEQGVANFSEEITDFNEQDAGWTTKIGSGMDATMNLGSSGDSTLGSFLGRPTRIADFSWVVDQPFFEKFDPWSLFLNDPRVAEKIANYELYRSKLHVKMVISGTGFHYGRALVSYNPYSGYDDITVERNFLQQDLVAASQKPHFFLNPTNNTGGQLDLPFFWPENYLSLSSSNRNSLGEMFIKSFSNLQHANEGNDPVNITVYAWASDVELTMPTSLTTL
Sogarnavirus	*Britarnavirus 3*	Marine RNA virus BC-3	MG584189	BC-3	88	EGSAAATEQITAFSDQEAGWTTCIKGGSDATMNLSSNSDSDLGNFLERPIRIHEAPWIMGQPLFTTFNPWVDFMSNPRVKEKIAHYELLRMNLHVKFVISGTGFHYGRALASYNPYLFDEITIQRNYLDVDLVQASQKPHIFLNPTTNSGGQLDLPYFYHKNYMSLSEFDSTRMGDITLKSFDVLRHANGGDDPVTVTAYAWASDVVLTMPTSLT
Sogarnavirus	*Chaetarnavirus 2*	Chaetoceros species RNA virus 02	AB639040	CspRNAV2	70	DSNSPSGGAYSVSKAAEQISTQNVHFVDGDTPWSYDISSSPDVTTQLAGFSDAELGSFLGRPIKIKEFQWTPESTRLFEVFNPWTEFFSNADVLEKINRYRNLRCNLRMKMLINGNSFYYGRALVSYNPYLTNDSVTLNRAFFEQDLVGASQKPHFMLDPTTSQGGEMLLPFLWPENFLDITQPNWTADMGRVTIHDFDILRHANGGTDPITVTVFVWAEDVVLSVPTTVQAQ
Sogarnavirus	*Chaetenuissarnavirus II*	Chaetoceros tenuissimus RNA virus type II	AB971661	CtenRNAVII	N/A	N/A
Sogarnavirus	*Jericarnavirus A*	Marine RNA virus JP-A	EF198241	JP-A	92	NSEGDTGFSVSKVDRESAAQNVHFVDGDTPWSYDIKANADETTKLAAFTDAELGDFLSRPIKIKEYQWTPGVALSAPRFNPWREFFNNSDVLDKINRYRNLRCNLRMKVLVNGNSFYYGRALLTYNPYIQNDEVTVNRTFIEEDLIQASQKPHLLLDPTTSQGGEMLLPFIWPENYLNITVAGWNANMGEVDIHDFDVLQHANGGTDPISITIFCWAENLTLAIPTTAQVS
Sogarnavirus	*Palmarnavirus 156*	Marine RNA virus PAL156	KT727024	PAL156	89	GTSQKPGTTNLTQEITAFSDQSAGWTTTIGSGSDPTMDLAATVDSDLGNFLGRPVRIAEYNWGVGSGFFERFNPWREFLNDERVKEKTAHFELYRSKLHVKMVVSGTGFHYGRALVSYNPLYGRDDVTIQRSFLQSDLVAASQKPHFFINPTTNAGGQLDLPFFYPDNYLSLTRGTDAQEMGEITIKSFDSLRHANGGTGNVTVTVYAWASDVVLAMPTSVNLL

Table S2. Related *Marnaviridae* virus species used for AlphaFold2 and the average pLDDT scores.

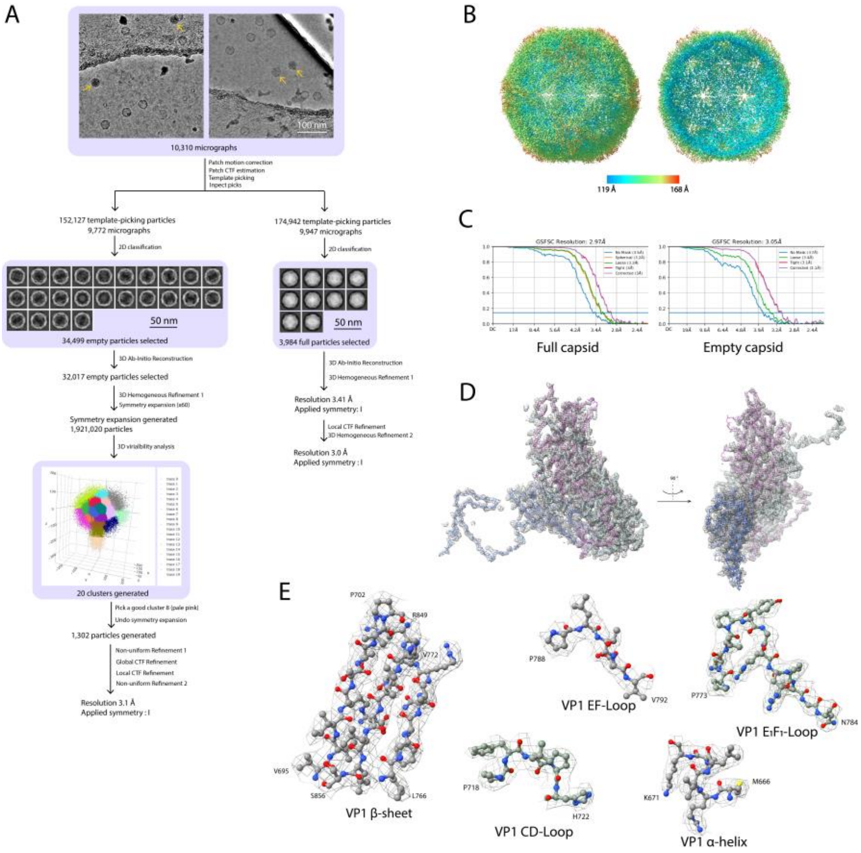

Figure S1. Summary of the cryo-EM data analysis. A) Flowchart of the CsfrRNAV capsid data analyses of the cryo-EM micrographs using cryoSPARC. Yellow arrows point out full CsfrRNAV particles in the cryo-EM micrographs. B) Overall 3D reconstruction of the full CsfrRNAV capsid. Left: The overall capsid map. Right: The back half of the capsid shows the interior features. Capsid is colored from blue to red according to its calculated radius. C) The gold standard FSC resolution curves of both full and empty particles in icosahedral (I) symmetry. The resolutions of these cryo-EM models are estimated as 3.0 (full) and 3.1 (empty) Å (FSC cutoff = 0.143). D) The cryo-EM map and the backbone fitting with the atomic models of the capsid VP1 (pink), VP2 (blue), and VP3 (light green). E) Representative β-sheet, loops and α-helix show the refined side chains in full capsid of CsfrRNAV.

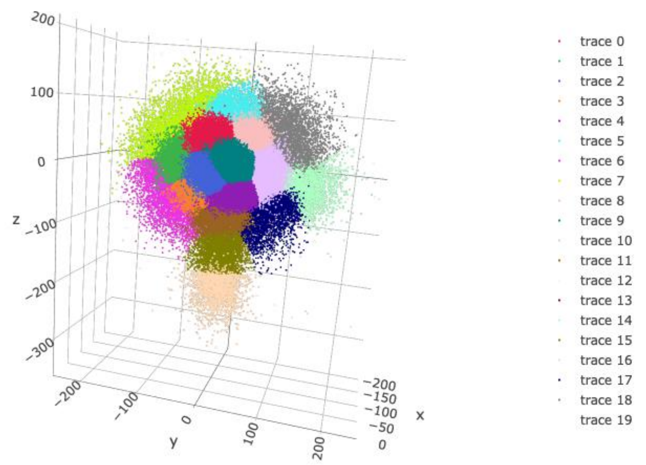

Figure S2. 3D variability display. The 3D variability display performed in cryoSPARC 3.3.2. The symmetry expanded empty particles are sorted into 20 clusters in total. Only the map in cluster 8 (pale pink) shows clear secondary structure and therefore is used for further 3D refinement.

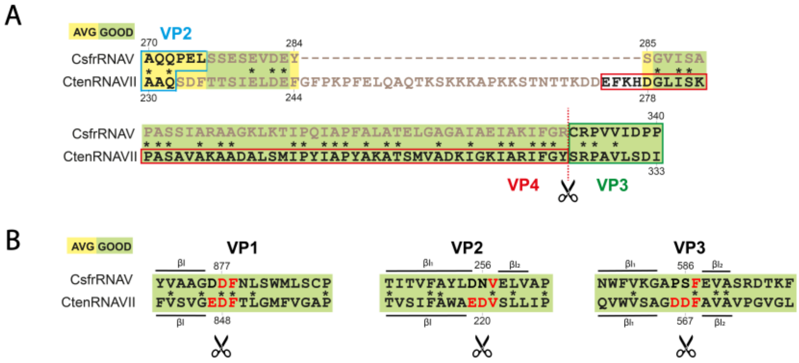

Figure S3. Self-cleavage sites and partial sequence alignments of CsfrRNAV and CtenRNAVII. AVG: average sequence identities. GOOD: good sequence identities. * Identical residues. A) Partial sequence alignment result and the putative VP4 VP3 proteolytic site in CsfrRNAV and CtenRNAVII. Blue, green, and red boxes represent parts of VP2, VP3, and VP4, respectively. Red dashed line represents the proteolytic site between VP4 and VP3. Modeled residues are in black. Unmodeled residues are in gray. B) Putative self-cleavage sites in the VP1, VP2, and VP3 of CsfrRNAV and CtenRNAVII. Residues that are potentially involved in self-cleavage in each VP are colored red. Sequences are aligned using the T-COFFEE (version 11.00) server [59].

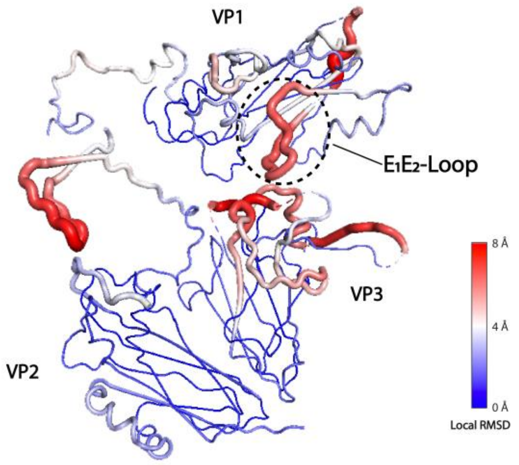

Figure S4. RMSD per residue for VP1–3 (CsfrRNAV vs CtenRNAVII). Residues are highlighted from blue to red, showing low to high RMSD values. VP2 does not show apparent structural differences apart from the N-terminal arm. VP3 shows some structural differences in the surface loops adjacent to the VP1 E_1E_2-Loop.

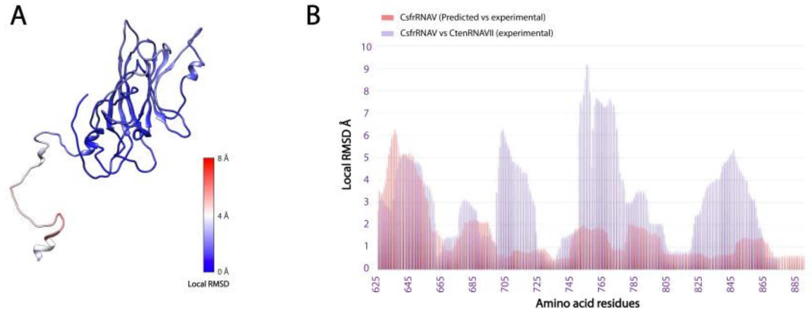

Figure S5. AlphaFold2 structural prediction error (predicted vs. experimental) and VP1 structural differences between two representative *Marnaviridae* viruses (CsfrRNAV vs. CtenRNAVII). A) The structural prediction error per residue between the experimental and predicted CsfrRNAV VP1s. The residues are colored from low to high deviations (blue to red) by the attribute of their local RMSD. B) The overall comparison between experimental CsfrRNAV VP1 and CtenRNAVII VP1 structural differences (purple) and the AlphaFold2 prediction errors (red), which were calculated using CsfrRNAV VP1 atomic model as a reference.

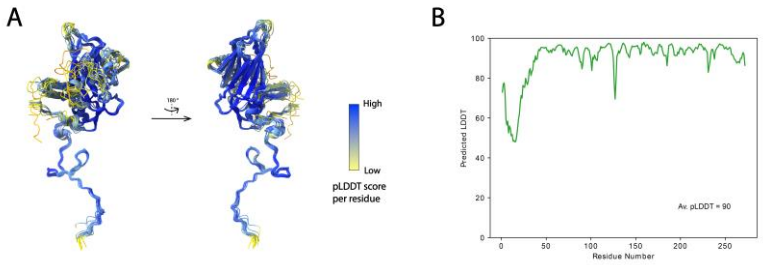

Figure S6. Superimposition of the predicted *Marnaviridae* viruses VP1s and the representative residue pLDDT score plot. A) Superimposition of VP1s from all 16 predicted *Marnaviridae* viruses. All models are colored according to their pLDDT scores by residue from yellow (backbone modeled well) to blue (high accuracy).

B) Predicted VP1 model of a locarnavirus (JN661160) is shown as an example plot of pLDDT score per residue.
